# Prediction of postoperative refractive astigmatism before toric intraocular lens implantation

**DOI:** 10.1186/s12886-021-01959-7

**Published:** 2021-05-08

**Authors:** Atsushi Kawahara

**Affiliations:** San Ai Eye Clinic, 1-3-28, Yamauchi, Okinawa, Japan

**Keywords:** Astigmatism, Refractive astigmatism, Corneal astigmatism, Toric intraocular lens, Cataract surgery

## Abstract

**Background:**

To determine the preoperative factors influencing refractive astigmatism after cataract surgery for astigmatism correction by toric intraocular lens (IOL) implantation and to evaluate the prediction model using these factors.

**Methods:**

Prospective, observational case series. The right eyes of forty consecutive patients with preoperative corneal astigmatism of the total cornea of 1.5 diopters (D) or more in magnitude and scheduled for implantation of a non-toric IOL during cataract surgery with a 2.4-mm temporal clear corneal incision were examined prospectively. The vertical/horizontal astigmatism component (J0) and oblique astigmatism component (J45) of refractive and corneal astigmatism were converted using power vector analysis. Multivariate regression analysis was performed with refractive astigmatism at three months postoperatively as the dependent variable, and preoperative parameters including age, sex, refractive astigmatism, corneal astigmatism, sphere, spherical equivalent, intraocular pressure, corneal thickness, anterior chamber depth, lens thickness, lens positions (tilt and decentration), axial length, and corneal higher order aberrations as independent variables. The root mean square (RMS) errors were calculated to express the regression model fit.

**Results:**

The regression model for the J0 component was $Postoperative\kern0.34em refractive\kern0.2em J0=1.05\times Coneal\kern0.2em J0-0.14$ (*R*^2^ = 0.96, *P* < 0.001). The model for the J45 component was $Postoperative\kern0.34em refractive\kern0.2em J45=0.68\times Coneal\kern0.2em J45+0.19\times Preoperative\kern0.34em refractive\kern0.2em J45-0.06$ (*R*^2^ = 0.72, *P* < 0.001). The mean RMS errors for preoperative corneal astigmatism alone and the multivariate model were 0.58 D and 0.46 D, respectively. There was a statistically significant difference between them (*P* = 0.02).

**Conclusions:**

Refractive astigmatism after implantation of a toric IOL can be predicted by the regression model more accurately than by corneal astigmatism alone. However, the prediction of oblique astigmatism remains a challenge.

## Background

In recent years, the goal of cataract surgery has shifted from improving postoperative corrected visual acuity to improving postoperative uncorrected visual acuity. In order to achieve this goal, it is necessary to eliminate postoperative residual astigmatism, and a toric intraocular lens (IOL) has become widely used. The correction of astigmatism with toric IOLs is based on the assumption that all astigmatism originates from the cornea and lens. However, it is known that the difference between postoperative refractive and corneal astigmatism is not nil [1, 2]. Postoperative refractive astigmatism is thought to include lens capsule (and/or IOL) tilt and decentration, and unknown ocular components in addition to corneal astigmatism. Therefore, it is necessary to reconsider which parameters predict postoperative refractive astigmatism in eyes indicated for toric IOLs.

Three observational studies showed that postoperative refractive and preoperative corneal astigmatism had a significant correlation in pseudophakic eyes with non-toric IOLs, and preoperative corneal astigmatism was the most significant predictor in multivariate regression analysis [3–5]. However, their correlations were assessed without considering the power of corneal astigmatism. The purpose of our study was to determine the predictors of postoperative refractive astigmatism in eyes with corneal astigmatism before cataract surgery for astigmatism correction by toric IOL implantation. To our knowledge, this study is the first study to assess the correlation between postoperative refractive astigmatism and preoperative parameters in pseudophakic eyes with non-toric IOLs, including the eye with an indication of toric IOL implantation.

## Methods

### Subjects

Consecutive patients who were scheduled for routine cataract surgery at the San Ai Eye Clinic between October 1, 2019 and September 30, 2020 were screened for possible inclusion in the study. The inclusion criteria were right eyes with preoperative total corneal astigmatism of 1.5 diopters (D) or more in magnitude and scheduled for phacoemulsification with implantation of non-toric IOLs. The exclusion criteria were eyes with any pathology of the cornea, optic nerve or macula; eyes with a lens nucleus harder than grade 4 [6]; eyes with poor mydriasis (< 4.5mm); eyes with a possible zonular dehiscence or pseudoexfoliation; eyes with a history of surgery or inflammation; and patients who wanted implantation of toric IOLs.

After topical anesthesia with 4 % lidocaine hydrochloride, coaxial phacoemulsification was performed through a 2.4 mm temporal clear corneal single-plane incision at the 9 o’clock position in the right eye. A side-port incision also created with the left hand in a forearm position comfortable for a right-handed surgeon. Single-piece acrylic non-toric IOLs were implanted in the capsular bag through an unenlarged temporal incision. All incisions were hydrated to aid closure of the incision. No eye required sutures. Spherical equivalent (SE) power of the IOL meeting the focal distance desired by the patient (emmetropia or mild myopia) was calculated preoperatively using the SRK/T formula. Two surgeons (AK and DK) performed all cataract surgeries.

### Outcome Measures

Except for the examining equipments and the timing of the examinations, we performed the same as in the previous study [3]. All eligible patients underwent ocular examinations preoperatively and at three months postoperatively, and data for corrected distance visual acuity (CDVA), refractive astigmatism, corneal astigmatism (total corneal astigmatism), sphere, SE, intraocular pressure (IOP), corneal thickness, anterior chamber depth, lens thickness, lens positions (tilt and decentration), axial length, and corneal higher order aberrations (HOAs) were collected. CDVA was examined using decimal charts, and this was converted to the logarithm of minimal angle of resolution (logMAR) scale. Objective refraction was measured using an autokerato/refractometer (ARK-530 A; Nidek, Gamagori, Japan). SE value was determined as the sum of the spherical power and half of the cylindrical power. IOP was measured using a non-contact tonometer (NT-4000; Nidek). Corneal astigmatism, corneal thickness, anterior chamber depth, lens thickness, lens positions (tilt and decentration), and corneal HOAs were examined using a swept-source optical coherence tomography (OCT; CASIA2, Tomey, Nagoya, Japan). The CASIA2 performs a three-dimensional analysis with sixteen different angles of anterior-segment OCT images and then automatically produces the measurements. The degree of lens tilt and decentration was evaluated in absolute values relative to the visual axis. Corneal HOAs were calculated by Fourier harmonic transformation. The refractive powers of the total cornea are transformed into four trigonometric components: the SE power, the first-order asymmetry, the second-order regular astigmatism, and the third and higher order irregularity. The asymmetry and third and higher order irregularity are defined as HOAs [7]. The repeatability and reproducibility of these corneal and anterior segment measurements were confirmed [8]. Axial length was measured using the IOLMaster 500 (Carl Zeiss Meditec AG, Jena, Germany) biometry. Information on age and sex were also extracted preoperatively.

Refractive and corneal astigmatism were expressed as a negative value and their values were converted to power vector components as described by Thibos et al. [9]. This analysis expresses the vertical (90°)/horizontal (180°) astigmatism component as J0, and the oblique astigmatism component (45° and 135°) as J45. Manifest refractions in conventional script notation (Sphere, Cylinder × Axis) were converted to power vectors by the following formulas:

$$ J0=-\left( Cylinder/2\right)\times \cos \left(2\times Axis\right) $$

$$ J45=-\left( Cylinder/2\right)\times \sin \left(2\times Axis\right) $$

In this representation, as astigmatism is represented in rectangular vector form, conventional scalar methods can be applied to each vector component, which simplifies the mathematical and statistical analysis of astigmatism.

### Statistical analysis

Statistical analysis was performed in much the same way as in the previous study [3].　Predictions of the postoperative refractive astigmatic components (J0 and J45) were determined by multivariate linear regression analysis. Multivariate regression analysis was performed with postoperative refractive J0 and J45 as the dependent variable, and preoperative parameters including age, sex, refractive J0 and J45, corneal J0 and J45, sphere, SE, IOP, corneal thickness, anterior chamber depth, lens thickness, lens positions (tilt and decentration), axial length, and corneal HOAs as independent variables. In the multivariate analysis, variables with a partial regression coefficient of P value > 0.10 were removed using the stepwise method. Variance inflation factors were calculated to assess multicollinearity. Variables with a variance inflation factor of more than ten were considered to have excessive collinearity and were excluded. Residual errors, which were differences between the measured and predicted values in the regression analysis of postoperative refractive J0 and J45, were also calculated. For residual analysis, independence of residual errors was assessed using the Durbin-Watson statistic, and heteroscedasticity was assessed using the Breusch-Pagan test. A Durbin-Watson statistic in the range of one and a half to two and a half indicates independence. Data were analyzed with BellCurve for Excel, version 3.21 (Social Survey Research Information Co., Ltd., Tokyo, Japan), with a P value < 0.05 considered statistically significant.

The root mean square (RMS) error was calculated to express the regression model fit in the familiar unit of diopters. The RMS error for each eye was as follows:

$$ RMS\kern0.2em error=2\times \sqrt{{\left(J{0}_{Measured}-J{0}_{Predicted}\right)}^2+{\left(\left(J{45}_{Measured}-J{45}_{Predicted}\right)\right)}^2} $$

The RMS error is appropriate for assessing standard least squares regression, which minimizes the squared error. Comparison of the RMS errors between preoperative corneal astigmatism alone and the multivariate model were performed using the Mann-Whitney U test.

## Results

All forty enrolled eyes completed the scheduled examinations. No eye had any perioperative complications. Table 1 shows the mean refractive and corneal astigmatism. The mean vector of preoperative and postoperative refractive astigmatism, and preoperative and postoperative corneal astigmatism was 1.24 D axis 180.0°, 0.89 D axis 0.0°, 0.59 D axis 180.0°, and 0.54 D axis 180.0°, respectively. For both refractive and corneal astigmatism, the mean of the absolute values of J0 is larger than the mean of the absolute values of J45, indicating less oblique astigmatism. The magnitude of corneal astigmatism was virtually unchanged between pre- and post-operatively, and refractive astigmatism was reduced, indicating that cataract surgery had eliminated lens astigmatism. Table 2 shows the variables for multivariate analysis other than astigmatism, although CDVA is not an independent variable.

**Table 1:** Refractive and corneal astigmatism

	Preoperative		Postoperative	
	Mean ± SD	Range	Mean ± SD	Range
Refractive astigmatism (D)	-2.42 ± 1.09	-5.50--0.75	-2.01 ± 0.86	-5.00--1.00
J0 component (D)	-0.62 ± 1.10	-2.55-1.62	-0.44 ± 0.94	-2.49-1.37
(The absolute value)	(1.11 ± 0.59)	(0.12-2.55)	(0.93 ± 0.48)	(0.09-2.49)
J45 component (D)	0.04 ± 0.41	-1.03-1.15	-0.03 ± 0.32	-0.86-0.49
(The absolute value)	(0.31 ± 0.28)	(0.00-1.15)	(0.26 ± 0.19)	(0.00-0.86)
Corneal astigmatism (D)*	-1.88 ± 0.57	-4.40--1.50	-1.79 ± 0.72	-4.30--0.90
J0 component (D)	-0.29 ± 0.88	-2.18-1.18	-0.27 ± 0.87	-2.14-1.20
(The absolute value)	(0.86 ± 0.34)	(0.21-2.18)	(0.82 ± 0.40)	(0.03-2.14)
J45 component (D)	0.03 ± 0.31	-0.52-0.72	0.01 ± 0.31	-0.63-0.62
(The absolute value)	(0.26 ± 0.18)	(0.00-0.72)	(0.26 ± 0.17)	(0.00-0.63)

*D* diopter, *J0* vertical/horizontal astigmatism component, *J45* oblique astigmatism component

*Corneal astigmatism of the total cornea

**Table 2:** Patient characteristics and measurements

Parameter	Preoperative		Postoperative	
	Mean ± SD	Range	Mean ± SD	Range
CDVA (logMAR)	0.14 ± 0.15	-0.18-0.52	-0.10 ± 0.07	-0.18-0.10
Age (years)	70.9 ± 8.8	46-87		
Sex (men/women)	21/19			
Sphere (D)	0.65 ± 3.79	-18.25-5.25	0.49 ± 0.92	-2.50-2.25
SE (D)	-0.56 ± 3.80	-19.50-3.75	-0.52 ± 0.85	-3.13-0.38
IOP (mmHg)	14.8 ± 2.6	10.0-22.0	12.8 ± 3.0	7.0-21.0
Corneal thickness (μm)	532 ± 32	468-606	533 ± 32	470-607
Anterior chamber depth (mm)	2.65 ± 0.46	1.46-3.41	4.24 ± 0.30	3.47-4.81
Lens thickness (mm)	4.65 ± 0.36	3.62-5.36		
Lens tilt (degree)	4.58 ± 1.34	0.80-6.90		
Lens decentration (mm)	0.15 ± 0.08	0.03-0.32		
Axial length (mm)	23.79 ± 1.53	21.44-29.67	23.70 ± 1.53	21.35-29.57
Corneal HOAs (μm)	0.27 ± 0.08	0.15-0.56	0.27 ± 0.07	0.14-0.49

*CDVA* corrected distance visual acuity, *logMAR* logarithm of minimal angle of resolution, *D* diopter, *SE* spherical equivalent, *IOP* intraocular pressure, *HOAs* higher order aberrations

After a stepwise multivariable regression analysis in postoperative refractive J0, preoperative corneal J0 was found to be an independent significant predictor (Table 3). The regression equation of postoperative refractive J0 was as follows:

**Table 3:** Multivariate regression analysis of postoperative refractive J0

Preoperative parameter	Partial regression coefficient	Standard partial regression coefficient	P value
Corneal J0	1.05	0.98	< 0.001
Intercept	-0.14		< 0.001

*J0* = vertical/horizontal astigmatism component

R^2^ = 0.96, P < 0.001

$$ Posoperative\kern0.2em J0=1.05\times Coneal\kern0.2em J0-0.14\kern0.2em \left({R}^2=0.96,\kern0.2em P<0.001\right) $$

In postoperative refractive J45, preoperative corneal and refractive J45 were found to be independent predictors (Table 4). The regression equation of postoperative refractive J45 was as follows:

**Table 4:** Multivariate regression analysis of postoperative refractive J45

Preoperative parameter	Partial regression coefficient	Standard partial regression coefficient	P value
Corneal J45	0.68	0.66	< 0.001
Refractive J45	0.19	0.25	0.045
Intercept	-0.06		0.057

*J45* = oblique astigmatism component

R^2^= 0.72, P < 0.001

$$ Postoperative\kern0.2em J45=0.68\times Coneal\kern0.2em J45+0.19\times Preoperative\kern0.34em refractive\kern0.2em J45-0.06\kern0.2em \left({R}^2=0.72,P<0.001\right) $$

There were no problems in either multicollinearity or residual analysis in both regression equations.

The mean RMS errors for preoperative corneal astigmatism alone and the multivariate models were 0.58 ± 0.25 D and 0.46 ± 0.25 D, respectively. The mean RMS error of the multivariate model was statistically significantly lower than that of corneal astigmatism alone (*P* = 0.02).

## Discussion

Multivariate regression analysis revealed that preoperative astigmatism was significantly correlated with postoperative refractive astigmatism in both the vertical/horizontal and oblique components. In the respective multivariate regression analyses, the standard partial regression coefficients of preoperative corneal astigmatism gave the large and maximum absolute values. The standard partial regression coefficients describe what the partial regression coefficients would equal if all variables had the same standard deviation. The magnitude of the standard partial regression coefficients shows the relative importance of different variables. These results suggest that preoperative corneal astigmatism is the most significant predictor of postoperative refractive astigmatism. In terms of R^2^ values, preoperative corneal astigmatism was largely predictive of postoperative refractive vertical/horizontal astigmatism. As for the oblique astigmatism, preoperative corneal and refractive astigmatism predicted postoperative refractive oblique astigmatism. It is possible that both refractive and corneal astigmatism should be considered when predicting postoperative refractive astigmatism as refractive astigmatism may contain real, independent information about both the anterior and posterior cornea not reflected in keratometry. However, the R^2^ value of the oblique astigmatism was less than the vertical/horizontal astigmatism. Taken together, the findings of the current study demonstrated that corneal astigmatism is the most significant predictor of postoperative refractive astigmatism, even in eyes with a corneal astigmatic error, and postoperative with-the-rule or against-the-rule refractive astigmatism can be independently predicted using corneal astigmatism, although the prediction of oblique astigmatism remains a challenge.

Observational studies demonstrated that preoperative corneal astigmatism was the most significant predictor in multivariate regression analysis, although the power of corneal astigmatism was not considered [3–5]. In addition, Leffler et al. reported that refractive astigmatism after cataract surgery and preoperative corneal astigmatism were correlated (R^2^ values: 0.51 and 0.05 for J0 and J45, respectively) [4]. Similar results have been addressed elsewhere [3, 5]. In contrast, the present study demonstrated that postoperative refractive and preoperative corneal and refractive astigmatism had a better correlation (R^2^ values: 0.96 and 0.72 for J0 and J45, respectively). This was probably due to the smaller incision width and/or the evaluation of the total cornea using swept-source anterior-segment OCT in our study. The regression equations obtained in this study allows the refractive astigmatism after cataract surgery to be estimated. Toric IOL power could subsequently be derived by transformation from the refractive to the IOL plane. These findings should be applicable to left eyes, although only right eyes were analyzed in this study. The reason for this is that refractive astigmatism [10–12], corneal curvature [13–16], and pupil center [17] demonstrate mirror interocular symmetry about the mid-sagittal plane. Further, the multivariate models improved the RMS error using only corneal astigmatism. Accordingly, we believe that it is appropriate to use the regression model for evaluation prior to cataract surgery using toric IOL implantation.

A potential limitation of the present study is the small number of cases. However, there is no statistical problem as multivariate linear regression equations in this study have one or two independent variables [18]. On the other hand, a larger number of cases would be needed to improve the accuracy of the regression model.

## Conclusions

This study showed that corneal astigmatism is the strongest correlate of postoperative refractive astigmatism in eyes with an indication of toric IOL implantation, but that refractive astigmatism can be predicted by the regression equation more accurately than by preoperative corneal astigmatism alone. However, the oblique astigmatism was not yet fully predicted. These findings added new insights to the prediction of postoperative astigmatism before toric IOL implantation, but further studies are needed to provide better predictions, especially in terms of oblique astigmatism.

## Data Availability

The datasets used and analyzed for the present study can be obtained from the corresponding author on reasonable request.

## Abbreviations

IOL: Intraocular lens
D: Diopters
SE: Spherical equivalent
CDVA: Corrected distance visual acuity
IOP: Intraocular pressure
HOAs: Higher order aberrations
logMAR: Logarithm of minimal angle of resolution
OCT: Optical coherence tomography
J0: Vertical/horizontal astigmatism component
J45: Oblique astigmatism component
RMS: Root mean square
